# Dataset of pharmaceuticals and personal care products in a Mediterranean coastal wetland

**DOI:** 10.1016/j.dib.2021.106934

**Published:** 2021-03-15

**Authors:** Daniele Sadutto, Vicente Andreu, Timo Ilo, Jarkko Akkanen, Yolanda Picó

**Affiliations:** a Research Center on Desertification (CIDE), Environmental and Food Safety Research Group of the University of Valencia (SAMA-UV), CSIC-UV-GV, Moncada-Naquera Road km 4.5, 46113 Moncada, Valencia, Spain; b Department of Environmental and Biological Sciences, University of Eastern Finland, P.O. Box 111, FI-80100 Joensuu, Finland

**Keywords:** Albufera natural park, PPCPs, Water, Soil, Sediment, Wastewater

## Abstract

The dataset provides information on Pharmaceutical and Personal Care Products (PPCPs) detected in the Albufera Natural Park (Valencia, Spain), a typical Mediterranean coastal wetland. These PPCPs constitute an important group of organic pollutants highly representative of the human impact.

The concentrations values measured in soil, sediment and water and the statistical relationship of contaminants between them and with the environmental parameters could help to understand their fate in different compartments. The data also reported the occurrence and removal efficiency (%) for each contaminant in ten wastewater treatment plants (WWTPs), located in the surrounding area. This dataset could provide an idea on the effectiveness of WWTP treatments and the capacity of released PPCPs to affect the ecosystem.

The extraction of analytes was based on solid-phase extraction (SPE) for water and solvent extraction followed by the previous SPE as clean-up for soil and sediment. Determination was carried out by high performance liquid chromatography tandem mass spectrometry (HPLC-MS/MS) with a triple-quadrupole.

The present dataset was analyzed within the article entitled: “*Pharmaceuticals and personal care products in a Mediterranean coastal wetland: Impact of anthropogenic and spatial factors and environmental risk assessment”*[1]*.*

## Specifications Table

**Table:** 

Subject	Pollution
Specific subject area	Pharmaceuticals and personal care products occurrence and fate in Mediterranean coastal wetlands.
Type of data	Table
How data were acquired	Raw data were acquired via HPLC-MS/MS. The instrument was an Infinity 1260 UHPLC system, coupled to mass spectrometry with a triple-quadrupole mass detector 6410 (QqQ-MS) from Agilent Technologies (Santa Clara, CA, USA). The ionization technique used was electrospray ionization (ESI).
Data format	Raw Analyzed Filtered
Parameters for data collection	The mobile phase consisted of 2.5 mmol L^−1^ NH_4_F in methanol (A) and 2.5 mmol L^−1^ NH_4_F in water (B) for negative mode and methanol and water with 0.1% formic acid in both solutions for positive mode. The other parameters are described in literature [2].
Description of data collection	Data were obtained analysing environmental samples (soil, sediment and water) collected in 44 sampling points of the Albufera Natural Park and in ten wastewater treatment plants (WWTPs), located in the surrounding area. The final value concentrations were achieved by HPLC-MS/MS analysis.
Data source location	Institution: University of Valencia, Research Center on Desertification (CIDE) City: Valencia Country: Spain Latitude and longitude (and GPS coordinates, if possible) for collected samples/data: All coordinates are specified in related research article [1]. Moreover, the virtual map of sampling points was reported in the interactive link: https://www.google.com/maps/d/viewer?mid=1ITmoW9a9uCVfqVZ08v25oNgMxjUFAMxn&ll=39.41350461328517%2C-0.41794440579224323&z=11
Data accessibility	Pico, Yolanda; Sadutto, Daniele; Andreu, Vicente; Ilo, Timo; Akkanen, Jarkko (2021), “Details on the Dataset of pharmaceuticals and personal care products in a Mediterranean coastal wetland”, Mendeley Data, V1, http://dx.doi.org/10.17632/zy2zg7dhgv.1
Related research article	Daniele Sadutto, Vicente Andreu, Timo Ilo, Jarkko Akkanen, Yolanda Picó. *Pharmaceuticals and personal care products in a Mediterranean coastal wetland: Impact of anthropogenic and spatial factors and environmental risk assessment*, Environmental Pollution, 2021, 271: p. 116353. https://doi.org/10.1016/j.envpol.2020.116353

## Value of the Data

• The contaminants monitoring of a Natural Park (Albufera) is needed to value the environmental risk and how it is related with anthropogenic pressures. In addition, statistical correlations show a relationship between studied PPCPs and the different matrices.
• National and international authorities, wastewater treatment plant managers and researchers can estimate removal, occurrence, transport and fate of PPCPs.
• The data of PPCPs occurrence in sediment, soil and water samples could serve as a knowledge base that allows the establishment of monitoring programs for the compounds that appear with a higher frequency and establish a better assessment of the risks that exist for this natural space.
• The concentrations on each sampling point may help to understand the geophysical distribution of these contaminants and can be compared with other studies.

## 1 Data Description

The presented data were collected in the Albufera Natural Park (Valencia, Spain), which is included in the list of international wetlands (RAMSAR) (since 1990), in the Natura 2000 network as a Special Protection Area for Birds (SPA) under the Birds Directive, and is considered a Site of Community Importance (SCI) under Habitats Directive. This area of about 21000 hectares (ha) included a coastal lagoon fed by streams, rivers and irrigation channels, a sandy shoreline belt, rice paddies, vegetables and orange orchards in its most external part, where 43 sampling points and ten wastewater treatment plants (WWTPs), that discharged into irrigation channels that eventually end up in the park, were monitored. Strong anthropic pressure has already been noted in the area due to the proximity of Valencia city and its metropolitan area [3]. The occurrence of 32 pharmaceutical and personal care products (PPCPs) was investigated from November 2016 to February 2017. Detailed information of each sampling site is provided in the related article. Tables 1–2 shows the concentration of PPCPs in influent (i) and effluent (e) wastewater samples with relative date of collection. While, the removal efficiency (%) for each compound was reported in Table 3. The occurrence of (32) water, (19) sediment and (33) soil samples was described in Tables 4–6. The data presented in Table 1, Table 2 and 4–6 are the average value of three method's replicates for each sample to check reproducibility. In the public repository (Mendeley Data) [4] have been published the additional tables (named table S. “n°”), that contain the results of each individual extraction as well as the average (Tables S1-S5) and the linearity (R^2^), LOD, LOQ and matrix effect (ME) for each contaminant (Table S6-S7). The Statistical correlations between the studied PPCPs in soils, sediment and water were described in Tables 7–9. At last, statistical correlations between contaminants and intrinsic characteristics of all matrices were schematized in Tables 10–12. The intrinsic characteristics considered were temperature, pH, total soluble salts, dissolved O_2_ and redox potential in water, organic matter, carbonates, lime, clay and sand, pH, electric conductivity and cationic exchange capacity in sediments and organic matter, carbonates, sodium, potassium, magnesium and calcium, pH, electric conductivity and cationic exchange capacity in soil.

**Table 1:** Concentration of PPCPs (ng L^−1^) in the influents (i) of the WWTPs*.

Compound	iAS	iCAT	iCAT	iPAL	iSAL	iPE	iPI	iPI	iPII	iPII	iPS	iQB	iSU
	25/11/2016	22/11/2016	13/01/2017	14/12/2016	13/01/2017	13/01/2017	22/11/2016	13/01/2017	22/11/2016	13/01/2017	25/11/2016	14/12/2016	25/11/2016
*Alprazolam*	2.30	n.d.**	2.42	4.03	1.43	3.01	3.82	4.34	4.23	4.01	2.33	7.12	1.91
*Atenolol*	132	5.63	63.5	113	22.9	162	236	335	154	244	82.0	257	174
*Atorvastatine*	83.1	2.65	134	157	27.2	59.7	148	278	166	231	63.0	274	61.0
*Bezafibrate*	5.22	0.91	52.3	1.62	1.21	3.02	29.5	35.0	26.3	21.0	0.32	31.0	3.12
*BPA*	562	37.1	4.27 × 10^3^	147	66.2	87.9	166	238	400	387	110	1.01 × 10^3^	92.0
*Butylparaben*	5.43	n.d.	18.4	19.2	1.82	n.d.	13.6	33.0	1.92	10.8	9.14	4.62	6.73
*Caffeine*	8.70 × 10^3^	9.78 × 10^3^	9.36 × 10^3^	6.08 × 10^3^	2.46 × 10^3^	5.24 × 10^3^	9.99 × 10^3^	1.32 × 10^3^	6.95 × 10^3^	8.36 × 10^3^	4.34 × 10^3^	18.07 × 10^3^	4.3 × 10^3^
*Chloramphenicol*	2.01	n.d.	n.d.	n.d.	n.d.	n.d.	n.d.	n.d.	n.d.	n.d.	n.d.	n.d.	1.41
*Clofibric acid*	n.d.	n.d.	n.d.	n.d.	n.d.	n.d.	n.d.	n.d.	n.d.	n.d.	n.d.	n.d.	n.d.
*Codeine*	68.4	1.12	52.2	7.01	10.0	50.2	52.1	133	80.9	93.8	47.5	71.8	72.0
*Diclofenac*	324	n.d.	290	303	68.0	123	324	380	386	320	327	348	275
*Enelapril*	379	n.d.	283	63.8	n.d.	n.d.	288	281	243	141	119	510	127
*Ethylparaben*	25.0	4.34	174	87.2	5.32	3.03	87.2	43.0	0.52	1.71	5.21	43.0	73.0
*Etoricoxib*	9.31	1.32	17.4	14.8	6.03	6.94	14.9	15.0	18.5	22.0	12.0	36.1	7.42
*Flufenamic acid*	126	0.84	78.0	75.2	26.4	86.7	136	175	178	205	48.9	415	95.3
*Furosemide*	411	n.d.	354	198	n.d.	268	444	700	557	598	266	927	310
*Ibuprofen*	11.0 × 10^3^	2.32 × 10^3^	12.60 × 10^3^	6.51 × 10^3^	2.57 × 10^3^	6.99 × 10^3^	7.97 × 10^3^	1.42 × 10^3^	7.79 × 10^3^	10.3 × 10^3^	5.58 × 10^3^	11.5 × 10^3^	57.6 × 10^3^
*Indomethacin*	6.03	n.d.	0.62	n.d.	1.03	n.d.	4.52	7.32	n.d.	3.82	n.d.	n.d.	0.72
*Lorazepam*	30.6	33.0	26.0	18.1	17.0	29.4	30.2	51.0	43.1	35.3	13.7	92.0	30.0
*Metformin*	190	3.62	338	323	47.9	286	256	410	260	321	206	319	135
*Methylparaben*	354	24.2	388	625	59.0	132	286	1.28 × 10^3^	36.5	24.8	164	115	610
*Naproxen*	2.56 × 10^3^	50.4	3.58 × 10^3^	632	570	1.84 × 10^3^	2.85 × 10^3^	3.38 × 10^3^	2.55 × 10^3^	2.85 × 10^3^	2.10 × 10^3^	3.02 × 10^3^	2.00 × 10^3^
*Omeprazole*	n.d.	n.d.	1.0	n.d.	n.d.	n.d.	n.d.	270	n.d.	n.d.	n.d.	n.d.	158
*Paracetamol*	2.83 × 10^3^	4.94 × 10^3^	5.92 × 10^3^	6.08 × 10^3^	3.15 × 10^3^	4.52 × 10^3^	4.34 × 10^3^	6.78 × 10^3^	1.86 × 10^3^	3.81 × 10^3^	5.50 × 10^3^	4.50 × 10^3^	5.35 × 10^3^
*Propylparaben*	188	11.7	395	288	39.9	319	373	627	111	224	290	134	275
*Salicylic acid*	779	167	2.01 × 10^3^	1.61 × 10^3^	250	394	1.41 × 10^3^	2.58 × 10^3^	237	187	730	1.49 × 10^3^	1.76 × 10^3^
*Simvastatin*	2.06 × 10^3^	n.d.	1.67 × 10^3^	1.30 × 10^3^	186	606	1.28 × 10^3^	2.02 × 10^3^	1.31 × 10^3^	2.03 × 10^3^	1.60 × 10^3^	2.05 × 10^3^	1.75 × 10^3^
*Thiamphenicol*	n.d.	n.d.	n.d.	n.d.	n.d.	n.d.	n.d.	n.d.	n.d.	n.d.	n.d.	n.d.	n.d.
*Tramadol*	569	153	551	297	138	419	587	859	681	750	220	1.05 × 10^3^	416
*Triclocarban*	0.74	0.42	n.d.	0.41	n.d.	0.53	1.72	1.51	1.12	0.63	0.91	0.82	0.71
*Triclosan*	102	40.4	99.5	77.0	50.0	n.d.	308	410	56.2	133	156	153	104
*Warfarin*	n.d.	n.d.	0.33	0.54	n.d.	0.45	n.d.	n.d.	1.15	n.d.	n.d.	0.21	n.d.

* WWTPs: Pinedo 1 (PI), Pinedo 2 (PII), Port de Catarroja (CAT), Quart - Benàger (QB), Sueca (SU), Perelló-Sueca (PS), Perellonet (PE), Palmar (PAL), Saler (SAL) and Albufera Sud (AS)

** n.d. = not detected

**Table 2:** Concentration of PPCPs (ng L^−1^) in the effluents (e) of the WWTPs*.

Compound	eAS	eCAT	eCAT	ePAL	eSAL	ePE	ePI	ePI	ePII	ePII	ePS	eQB	eSU
	25/11/2016	22/11/2016	13/01/2017	14/12/2016	13/01/2017	13/01/2017	22/11/2016	13/01/2017	22/11/2016	13/01/2017	25/11/2016	14/12/2016	25/11/2016
*Alprazolam*	3.20	n.d.**	n.d.	5.90	1.60	2.30	7.50	5.10	9.00	7.00	2.90	7.70	4.60
*Atenolol*	14.0	8.40	140	26.0	19.0	4.00	148	190	125	137	11.7	104	24.4
*Atorvastatine*	n.d.	n.d.	n.d.	n.d.	n.d.	n.d.	152	143	52.9	146	n.d.	19.0	15.7
*Bezafibrate*	1.80	0.50	0.30	0.40	2.00	1.00	29.6	35.1	6.90	20.4	0.50	20.4	12.3
*BPA*	93.0	19.0	n.d.	35.8	11.0	41.0	181	165	65.3	240	71.0	221	83.0
*Butylparaben*	0.40	n.d.	n.d.	n.d.	n.d.	n.d.	0.20	0.20	n.d.	1.50	n.d.	n.d.	n.d.
*Caffeine*	8.80	5.50	43.0	149	28.9	36.0	15.3	72.0	40.4	151	26.8	455	40.0
*Chloramphenicol*	n.d.	n.d.	n.d.	n.d.	n.d.	n.d.	n.d.	n.d.	n.d.	n.d.	n.d.	n.d.	n.d.
*Clofibric acid*	n.d.	n.d.	n.d.	n.d.	n.d.	n.d.	n.d.	n.d.	n.d.	n.d.	n.d.	n.d.	n.d.
*Codeine*	14.1	1.40	0.20	7.10	3.50	7.30	50.1	76.1	53.6	96.7	24.5	32.8	31.0
*Diclofenac*	164	n.d.	n.d.	164	29.0	57.0	711	433	525	490	114	259	199
*Enelapril*	n.d.	n.d.	n.d.	n.d.	n.d.	n.d.	n.d.	83.0	n.d.	n.d.	n.d.	n.d.	n.d.
*Ethylparaben*	n.d.	n.d.	0.20	0.30	0.50	0.40	n.d.	0.50	n.d.	1.00	0.60	4.00	0.30
*Etoricoxib*	8.00	2.60	1.40	19.5	3.70	4.70	25.7	24.7	30.2	22.5	13.4	22.1	27.7
*Flufenamic acid*	137	0.40	n.d.	95.8	22.0	65.0	350	200	277	200	45.0	328	187
*Furosemide*	134	n.d.	n.d.	n.d.	2.00	30.0	904	550	552	630	66.1	427	313
*Ibuprofen*	n.d.	499	231	n.d.	n.d.	n.d.	n.d.	n.d.	n.d.	n.d.	n.d.	n.d.	n.d.
*Indomethacin*	n.d.	n.d.	n.d.	n.d.	n.d.	n.d.	n.d.	1.80	n.d.	7.90	n.d.	n.d.	n.d.
*Lorazepam*	18.1	n.d.	n.d.	42.9	8.60	17.0	61.3	54.4	63.1	57.1	28.0	46.6	36.5
*Metformin*	6.50	2.00	4.40	9.90	1.10	8.00	66.3	34.9	18.9	24.0	15.6	23.4	26.5
*Methylparaben*	20.6	n.d.	n.d.	13.6	5.60	4.60	29.1	40.0	20.2	35.0	16.9	30.1	28.7
*Naproxen*	23.0	n.d.	n.d.	26.3	n.d.	n.d.	75.4	n.d.	90.7	146	26.1	318	205
*Omeprazole*	n.d.	n.d.	n.d.	n.d.	n.d.	n.d.	30.0	14.0	25.6	23.0	4.00	16.0	n.d.
*Paracetamol*	11.3	2.00	19.0	34.8	49.0	39.0	18.1	37.0	16.6	15.4	24.1	37.0	70.0
*Propylparaben*	1.30	n.d.	1.70	5.30	4.10	5.00	5.80	10.0	3.70	8.70	3.00	6.20	2.30
*Salicylic acid*	1.40	58.4	108	380	310	248	207	345	205	325	124	580	212
*Simvastatin*	n.d.	245	80.0	n.d.	n.d.	n.d.	n.d.	35.0	n.d.	n.d.	n.d.	n.d.	n.d.
*Thiamphenicol*	n.d.	n.d.	n.d.	n.d.	n.d.	n.d.	n.d.	9.00	n.d.	14.8	n.d.	n.d.	n.d.
*Tramadol*	470	n.d.	3.50	576	120	467	1.10 × 10^3^	994	1.28 × 10^3^	1.30 × 10^3^	273	11.6 × 10^3^	666
*Triclocarban*	n.d.	0.40	n.d.	0.30	0.10	0.10	0.60	0.50	n.d.	0.4	1.20	0.40	0.50
*Triclosan*	24.0	n.d.	n.d.	22.6	4.30	n.d.	29.2	20.3	10.4	7.4	n.d.	14.0	26.3
*Warfarin*	0.50	n.d.	n.d.	0.30	n.d.	n.d.	n.d.	n.d.	0.80	0.7	n.d.	0.10	0.40

** WWTPs: Pinedo 1 (PI), Pinedo 2 (PII), Port de Catarroja (CAT), Quart - Benàger (QB), Sueca (SU), Perelló-Sueca (PS), Perellonet (PE), Palmar (PAL), Saler (SAL) and Albufera Sud (AS)

** n.d. = not detected

**Table 3:** Removal efficiency (%) for each PPCPs in each WWTPs: Pinedo 1 (PI), Pinedo 2 (PII), Port de Catarroja (CAT), Quart - Benàger (QB), Sueca (SU), Perelló-Sueca (PS), Perellonet (PE), Palmar (PAL), Saler (SAL) and Albufera Sud (AS).

Compound	AS	CAT	CAT	PAL	EPE	PI	PI	PII	PII	PS	EQB	SAL	SU
	25/11/2016	22/11/2016	13/01/2017	14/12/2016	13/01/2017	22/11/2016	13/01/2017	22/11/2016	13/01/2017	25/11/2016	14/12/2016	13/01/2017	25/11/2016
*Alprazolam*	−37	/*	100	−47	22	−99	−18	−115	−75	−26	−9	−12	−146
*Atenolol*	89	−51	−120	77	98	37	43	19	44	86	60	17	86
*Atorvastatine*	100	100	100	100	100	−3	49	68	37	100	93	100	74
*Bezafibrate*	66	51	99	72	66	/	/	74	3	−67	34	−57	−291
*BPA*	83	49	100	76	53	−9	31	84	38	35	78	83	10
*Butylparaben*	93	/	100	100	/	98	99	100	86	100	100	100	100
*Caffeine*	100	100	100	98	99	100	99	99	98	99	97	99	99
*Chloramphenicol*	100	/	/	/	/	/	/	/	/	/	/	/	100
*Clofibric acid*	/	/	/	/	/	/	/	/	/	/	/	/	/
*Codeine*	79	−26	100	−1	85	4	43	34	−3	48	54	65	57
*Diclofenac*	49	/	100	46	54	−119	−14	−36	−53	65	26	57	28
*Enelapril*	100	/	100	100	/	100	70	100	100	100	100	/	100
*Ethylparaben*	100	100	100	100	87	100	99	100	42	89	91	90	100
*Etoricoxib*	14	−108	92	−31	32	−72	−65	−63	−2	−12	39	39	−275
*Flufenamic acid*	−8	48	100	−27	25	−158	−14	−56	2	8	21	17	−96
*Furosemide*	67	/	100	100	89	−103	21	1	−5	75	54	!	−1
*Ibuprofen*	100	79	98	100	100	100	100	100	100	100	100	100	100
*Indomethacin*	100	/	100	/	/	100	76	/	−108	/	/	100	100
*Lorazepam*	41	100	100	−137	42	−103	−7	−46	−62	−104	49	49	−22
*Metformin*	97	44	99	97	97	74	91	93	93	92	93	98	80
*Methylparaben*	94	100	100	98	96	90	97	45	−41	90	74	90	95
*Naproxen*	99	100	100	96	100	97	100	96	95	99	89	100	90
*Omeprazole*	/	/	100	/	/	!**	95	!	!	!	!	/	100
*Paracetamol*	100	100	100	99	99	100	99	99	100	100	99	98	99
*Propylparaben*	99	100	100	98	98	98	98	97	96	99	95	90	99
*Salicylic acid*	82	65	95	76	37	85	87	14	−74	83	61	−24	88
*Simvastatin*	100	!	95	100	100	100	98	100	100	100	100	100	100
*Thiamphenicol*	/	/	/	/	/	/	!	/	!	/	/	/	/
*Tramadol*	17	100	99	−94	−11	−89	−16	−88	−74	−24	−10	13	−60
*Triclocarban*	100	13	/	29	85	68	67	100	38	−31	49	!	26
*Triclosan*	76	100	100	71	/	91	95	82	94	100	91	91	75
*Warfarin*	!	/	100	39	100	/	/	26	!	/	71	/	!

* “/” means that compound was not detected in influent and effluent wastewater samples **“!” means that compound was detected only in effluent wastewater sample.

**Table 4:** Concentration of PPCPs (ng L^−1^) in water samples (W.n°) of the Albufera Natural Park, Valencia, Spain.

Compound	W.1	W.4	W.5	W.6	W.7	W.10	W.11	W.12	W.14	W.15	W.16	W.17	W.18	W.19	W.20	W.21	W.22	W.23	W.26	W.27	W.29	W.30	W.31	W.32	W.33	W.34	W.36	W.37	W.38	W.39	W.40	W.41	W.42
																																	
*Alprazolam*	10.0	4.00	n.d.*	6.00	n.d.	n.d.	n.d.	1.00	n.d.	n.d.	n.d.	n.d.	n.d.	n.d.	n.d.	n.d.	n.d.	n.d.	n.d.	n.d.	n.d.	2.00	n.d.	8.00	n.d.	n.d.	n.d.	n.d.	n.d.	n.d.	n.d.	n.d.	n.d.
*Atenolol*	320	114	n.d.	n.d.	n.d.	n.d.	n.d.	n.d.	n.d.	n.d.	62.0	n.d.	n.d.	n.d.	n.d.	n.d.	n.d.	78	n.d.	n.d.	n.d.	92.0	52.0	221	72.0	n.d.	n.d.	n.d.	n.d.	n.d.	n.d.	n.d.	n.d.
*Atorvastatin*	1.00	n.d.	n.d.	1.00	n.d.	n.d.	n.d.	n.d.	21.0	n.d.	n.d.	n.d.	n.d.	1.00	n.d.	n.d.	n.d.	n.d.	n.d.	n.d.	2.00	1.00	n.d.	21.0	1.00	21.0	1.00	21.0	n.d.	1.00	21.0	1.00	n.d.
*Bezafibrate*	79.0	63.0	n.d.	n.d.	n.d.	n.d.	n.d.	n.d.	n.d.	n.d.	n.d.	n.d.	n.d.	n.d.	n.d.	1.00	n.d.	2.00	n.d.	n.d.	n.d.	9.00	n.d.	75.0	n.d.	n.d.	n.d.	n.d.	n.d.	n.d.	n.d.	n.d.	n.d.
*Bisphenol A*	185	145	150	120	48.0	65.0	75.0	56.0	70.0	25.0	51.0	48.0	72.0	45.0	140	57.0	31.0	12.0	19.0	115	140	190	115	158	60.0	205	197	54.0	150	158	130	81.0	150
*Butylparaben*	n.d.	n.d.	70.0	70.0	45.0	n.d.	n.d.	n.d.	n.d.	40.0	n.d.	n.d.	40.0	45.0	n.d.	40.0	n.d.	n.d.	n.d.	70.0	n.d.	n.d.	70.0	n.d.	40.0	n.d.	71.0	n.d.	70.0	70.0	42.0	42.0	n.d.
*Caffeine*	152	28.0	62.0	22.0	137	56.0	11.0	14.0	291	76.0	152	21.0	134	101	121	219	40.0	668	20.0	43.0	138	541	103	272	157	66.0	173	93.0	141	127	120	16.0	18.0
*Chloramphenicol*	n.d.	n.d.	n.d.	50.0	n.d.	n.d.	n.d.	n.d.	n.d.	n.d.	n.d.	n.d.	39.0	40.0	n.d.	n.d.	n.d.	n.d.	n.d.	n.d.	n.d.	n.d.	n.d.	n.d.	40.0	n.d.	50.0	n.d.	n.d.	50.0	n.d.	n.d.	n.d.
*Clofibric acid*	76.0	77.0	76.0	76.0	n.d.	n.d.	n.d.	n.d.	n.d.	n.d.	n.d.	n.d.	n.d.	n.d.	77.0	n.d.	n.d.	n.d.	n.d.	77.0	76.0	75.0	76.0	76.0	n.d.	75.0	76.0	n.d.	76.0	76.0	75.0	n.d.	80.0
*Codeine*	154	27.0	n.d.	26.0	n.d.	n.d.	n.d.	n.d.	n.d.	n.d.	8.00	n.d.	n.d.	n.d.	7.00	16.0	n.d.	21.0	n.d.	n.d.	n.d.	36.0	30.0	84.0	12.0	n.d.	39.0	n.d.	n.d.	n.d.	n.d.	n.d.	n.d.
*Diclofenac*	169	70.0	50.0	115	60.0	52.0	75.0	55.0	n.d.	75.0	90.0	67.0	72.0	63.0	n.d.	75.0	65.0	103	75.0	n.d.	45.0	95.0	65.0	82.0	78.0	66.0	n.d.	104	45.0	35.0	72.0	35.0	30.0
*Enalapril*	n.d.	n.d.	n.d.	n.d.	n.d.	n.d.	n.d.	n.d.	n.d.	n.d.	4.00	n.d.	n.d.	n.d.	n.d.	n.d.	n.d.	n.d.	n.d.	n.d.	n.d.	n.d.	n.d.	n.d.	8.00	n.d.	n.d.	n.d.	n.d.	n.d.	n.d.	n.d.	n.d.
*Ethylparaben*	n.d.	n.d.	72.0	73.0	78.0	72.0	n.d.	82.0	n.d.	70.0	n.d.	70.0	70.0	70.0	n.d.	n.d.	n.d.	77.0	73.0	67.0	n.d.	75.0	66.0	67.0	80.0	79.0	70.0	76.0	65.0	n.d.	75.0	70.0	n.d.
*Etoricoxib*	18.0	4.00	3.00	8.00	1.00	n.d.	1.00	1.00	n.d.	n.d.	2.00	n.d.	n.d.	1.00	2.00	2.00	n.d.	2.00	n.d.	2.00	2.00	5.00	5.00	13.0	n.d.	n.d.	6.00	7.00	25.0	20.0	3.00	3.00	5.00
*Flufenamic Acid*	195	85.0	60.0	150	n.d.	1.00	n.d.	n.d.	1.00	2.00	4.00	2.00	n.d.	n.d.	61.0	7.00	1.00	15.0	n.d.	n.d.	n.d.	80.0	80.0	140	10.0	n.d.	n.d.	1.00	n.d.	n.d.	n.d.	n.d.	n.d.
*Furosemide*	n.d.	115	n.d.	74.0	48.0	n.d.	n.d.	n.d.	n.d.	70.0	90.0	n.d.	n.d.	n.d.	n.d.	n.d.	n.d.	65.0	n.d.	n.d.	n.d.	95.0	85.0	n.d.	n.d.	n.d.	n.d.	n.d.	n.d.	n.d.	n.d.	n.d.	n.d.
*Ibuprofen*	90.0	85.0	20.0	35.0	n.d.	n.d.	n.d.	n.d.	n.d.	n.d.	n.d.	n.d.	n.d.	n.d.	n.d.	n.d.	n.d.	n.d.	n.d.	n.d.	144	60.0	217	n.d.	n.d.	n.d.	90.0	n.d.	n.d.	43.0	n.d.	n.d.	n.d.
*Indomethacin*	56.0	n.d.	n.d.	n.d.	n.d.	n.d.	n.d.	n.d.	n.d.	n.d.	n.d.	25.0	n.d.	n.d.	n.d.	24.0	n.d.	n.d.	n.d.	n.d.	n.d.	n.d.	50.0	n.d.	25.0	n.d.	n.d.	n.d.	n.d.	n.d.	n.d.	n.d.	n.d.
*Lorazepam*	88.0	15.0	n.d.	15.0	n.d.	n.d.	n.d.	n.d.	n.d.	n.d.	18.0	n.d.	n.d.	n.d.	n.d.	6.00	n.d.	31.0	n.d.	n.d.	n.d.	23.0	12.0	70.0	1.00	n.d.	n.d.	2.00	n.d.	n.d.	n.d.	5.00	n.d.
*Metformin*	251	66.0	n.d.	19.0	20.0	4.00	3.00	5.00	23.0	15.0	114	26.0	171	3.00	14.0	55.0	2.00	131	375	24.0	25.0	186	70.0	93.0	57.0	18.0	22.0	33.0	94.0	40.0	40.0	6.00	19.0
*Methylparaben*	75.0	75.0	107	73.0	82.0	74.0	75.0	80.0	77.0	75.0	79.0	72.0	75.0	74.0	n.d.	75.0	n.d.	82.0	75.0	n.d.	70.0	84.0	75.0	78.0	75.0	73.0	78.0	76.0	73.0	n.d.	75.0	82.0	74.0
*Naproxen*	195	145	n.d.	105	n.d.	n.d.	n.d.	n.d.	115	n.d.	n.d.	n.d.	n.d.	n.d.	n.d.	117	114	208	111	n.d.	n.d.	209	225	178	160	n.d.	86.0	n.d.	n.d.	n.d.	113	115	n.d.
*Omeprazole*	n.d.	n.d.	n.d.	n.d.	n.d.	n.d.	n.d.	n.d.	n.d.	n.d.	n.d.	n.d.	n.d.	n.d.	n.d.	n.d.	n.d.	n.d.	n.d.	n.d.	n.d.	n.d.	n.d.	n.d.	n.d.	n.d.	n.d.	n.d.	n.d.	n.d.	n.d.	n.d.	n.d.
*Paracetamol*	26.0	n.d.	5.00	n.d.	32.0	14.0	11.0	17.0	82.0	28.0	18.0	n.d.	32.0	31.0	11.0	40.0	n.d.	168	n.d.	25.0	33.0	75.0	17.0	37.0	49.0	43.0	47.0	23.0	23.0	25.0	15.0	23.0	26.0
*Propylparaben*	n.d.	88.0	75.0	n.d.	38.0	33.0	32.0	35.0	33.0	32.0	30.0	30.0	30.0	30.0	75.0	35.0	30.0	33.0	30.0	90.0	75.0	75.0	75.0	135	31.0	n.d.	75.0	31.0	75.0	70.0	32.0	n.d.	90.0
*Salicylic Acid*	99.0	88.0	75.0	113	690	602	n.d.	n.d.	840	282	249	n.d.	298	391	294	239	208	751	340	106	521	713	75.0	161	230	686	166	592	240	858	386	301	204
*Simvastatin*	n.d.	n.d.	n.d.	n.d.	n.d.	n.d.	n.d.	n.d.	n.d.	n.d.	n.d.	n.d.	n.d.	n.d.	n.d.	n.d.	n.d.	n.d.	n.d.	n.d.	n.d.	n.d.	n.d.	n.d.	n.d.	n.d.	n.d.	n.d.	n.d.	n.d.	n.d.	n.d.	n.d.
*Thiamphenicol*	35.0	n.d.	n.d.	n.d.	n.d.	5.00	n.d.	1.00	n.d.	n.d.	5.00	n.d.	n.d.	n.d.	n.d.	n.d.	n.d.	5.0	n.d.	n.d.	n.d.	n.d.	n.d.	n.d.	n.d.	n.d.	n.d.	n.d.	n.d.	n.d.	n.d.	5.00	35.0
*Tramadol*	12.6 × 10^2^	197	1.00	523	n.d.	11.0	13.0	12.0	3.00	5.00	18.0	9.00	33.0	7.00	7.0	84.0	n.d.	106	n.d.	22.0	27.0	333	171	695	89.0	3.00	50.0	24.0	56.0	42.0	18.0	n.d.	5.00
*Triclocarban*	n.d.	15.0	13.0	n.d.	n.d.	2.00	n.d.	1.00	1.00	1.00	1.00	1.00	1.00	n.d.	n.d.	5.00	n.d.	1.00	n.d.	14.0	n.d.	1.00	13.0	n.d.	n.d.	n.d.	n.d.	1.00	13.0	13.0	15.0	1.00	15.0
*Triclosan*	65.0	28.0	n.d.	n.d.	n.d.	n.d.	44.0	50.0	60.0	65.0	n.d.	51.0	55.0	48.0	15.0	72.0	n.d.	52.0	44.0	n.d.	25.0	n.d.	52.0	56.0	50.0	48.0	50.0	n.d.	45.0	31.0	35.0	65.0	20.0
*Warfarin*	65.0	65.0	65.0	64.0	n.d.	45.0	n.d.	n.d.	n.d.	n.d.	45.0	n.d.	48.0	n.d.	65.0	45.0	n.d.	n.d.	45.0	n.d.	n.d.	65.0	70.0	70.0	45.0	65.0	n.d.	46.0	65.0	65.0	n.d.	46.0	n.d.

* n.d. = not detected

**Table 5:** Concentration of PPCPs (ng g^−1^) in sediment samples (S.n°) of the Albufera Natural Park, Valencia, Spain.

Compound	S.5	S.9	S.10	S.15	S.16	S.17	S.18	S.23	S.26	S.30	S.33	S.34	S.35	S.36	S.37	S.38	S.39	S.40	S.41
*Alprazolam*	n.d.*	n.d.	n.d.	n.d.	n.d.	n.d.	n.d.	n.d.	n.d.	n.d.	n.d.	n.d.	n.d.	n.d.	n.d.	n.d.	n.d.	n.d.	n.d.
*Atenolol*	n.d.	3.0	n.d.	n.d.	2.0	5.0	4.0	4.0	3.0	5.0	5.0	3.0	1.0	n.d.	1.0	4.0	n.d.	n.d.	16
*Atorvastatin*	n.d.	n.d.	n.d.	n.d.	n.d.	n.d.	n.d.	2.0	1.0	n.d.	21	1.0	21	n.d.	1.0	21	n.d.	1.0	21
*Bezafibrate*	n.d.	n.d.	n.d.	3.0	n.d.	n.d.	3.0	n.d.	n.d.	n.d.	n.d.	n.d.	n.d.	n.d.	n.d.	n.d.	3.0	3.0	n.d.
*Bisphenol A*	n.d.	15	20	5.0	14	7.0	10	21	7.0	20	18	20	2.0	n.d.	9.0	14	19	19	n.d.
*Butylparaben*	6.0	6.0	n.d.	6.0	7.0	n.d.	6.0	n.d.	n.d.	6.0	6.0	n.d.	6.0	n.d.	6.0	6.0	6.0	6.0	n.d.
*Caffeine*	6.0	9.0	8.0	10	7.0	5.0	8.0	8.0	6.0	7.0	6.0	9.0	6.0	4.0	5.0	4.0	6.0	10	7.0
*Chloramphenicol*	n.d.	4.0	n.d.	n.d.	n.d.	n.d.	n.d.	n.d.	n.d.	n.d.	n.d.	n.d.	n.d.	n.d.	n.d.	n.d.	n.d.	n.d.	n.d.
*Clofibric acid*	n.d.	5.0	n.d.	n.d.	n.d.	n.d.	5.0	5.0	5.0	n.d.	4.0	5.0	4.0	4.0	5.0	4.0	5.0	5.0	5.0
*Codeine*	n.d.	1.0	n.d.	n.d.	n.d.	n.d.	n.d.	n.d.	n.d.	n.d.	n.d.	1.0	n.d.	n.d.	n.d.	n.d.	n.d.	n.d.	n.d.
*Diclofenac*	3.0	3.0	8.0	8.0	10	6.0	7.0	5.0	n.d.	3.0	6.0	6.0	6.0	n.d.	5.0	3.0	8.0	4.0	4.0
*Enalapril*	n.d.	n.d.	n.d.	n.d.	n.d.	n.d.	n.d.	n.d.	n.d.	n.d.	n.d.	n.d.	n.d.	n.d.	n.d.	n.d.	n.d.	n.d.	n.d.
*Ethylparaben*	n.d.	17	15	n.d.	16	n.d.	n.d.	15	15	18	15	17	18	15	n.d.	15	17	15	18
*Etoricoxib*	1.0	n.d.	1.0	n.d.	n.d.	6.0	n.d.	1.0	1.0	1.0	1.0	1.0	n.d.	2.0	1.0	1.0	1.0	1.0	1.0
*Flufenamic Acid*	2.0	2.0	2.0	2.0	2.0	2.0	2.0	2.0	2.0	3.0	2.0	2.0	2.0	2.0	2.0	2.0	2.0	2.0	2.0
*Furosemide*	10	9.0	9.0	12	14	22	12	22	9.0	13	15	48	10	9.0	9.0	9.0	9.0	9.0	10.0
*Ibuprofen*	47	13	10 x 10^1^	24	22	30	35	70	22	7.0	23	29	63	n.d.	38	8.0	n.d.	28	4.0
*Indomethacin*	n.d.	n.d.	n.d.	n.d.	n.d.	n.d.	n.d.	n.d.	n.d.	n.d.	n.d.	n.d.	n.d.	n.d.	n.d.	n.d.	n.d.	n.d.	n.d.
*Lorazepam*	n.d.	n.d.	n.d.	n.d.	n.d.	n.d.	n.d.	n.d.	n.d.	n.d.	n.d.	n.d.	n.d.	n.d.	n.d.	n.d.	n.d.	n.d.	n.d.
*Metformin*	4.0	2.0	1.0	1.0	5.0	3.0	1.0	4.0	1.0	2.0	2.0	1.0	2.0	1.0	1.0	1.0	2.0	1.0	1.0
*Methylparaben*	n.d.	13	13	n.d.	14	n.d.	13	n.d.	17	18	18	18	19	n.d.	13	n.d.	14	14	n.d.
*Naproxen*	n.d.	n.d.	n.d.	2.0	n.d.	n.d.	n.d.	31	5.0	n.d.	8.0	n.d.	n.d.	2.0	4.0	7.0	7.0	10.0	n.d.
*Omeprazole*	n.d.	n.d.	n.d.	n.d.	n.d.	n.d.	n.d.	n.d.	n.d.	n.d.	1.0	n.d.	n.d.	n.d.	n.d.	n.d.	n.d.	n.d.	n.d.
*Paracetamol*	n.d.	6.0	n.d.	2.0	2.0	1.0	n.d.	n.d.	33	n.d.	1.0	n.d.	n.d.	2.0	n.d.	6.0	6.0	3.0	n.d.
*Propylparaben*	4.0	2.0	n.d.	2.0	n.d.	n.d.	2.0	4.0	n.d.	2.0	9.0	9.0	9.0	9.0	12	9.0	9.0	9.0	9.0
*Salicylic Acid*	21	18	25	32	28	24	23	23	19	23	18	23	19	19	16	15	18	21	27
*Simvastatin*	8.0	21	21	13	n.d.	29	12	n.d.	n.d.	17	n.d.	12	n.d.	n.d.	n.d.	18	n.d.	n.d.	4.0
*Thiamphenicol*	12	n.d.	11	9.0	10	9.0	11	11	12	14	n.d.	1.0	n.d.	n.d.	n.d.	n.d.	n.d.	1.0	n.d.
*Tramadol*	n.d.	n.d.	1.0	n.d.	1.0	2.0	2.0	2.0	n.d.	13	2.0	n.d.	n.d.	n.d.	n.d.	n.d.	n.d.	1.0	1.0
*Triclocarban*	n.d.	n.d.	n.d.	n.d.	n.d.	n.d.	n.d.	n.d.	n.d.	n.d.	7	9	15	n.d.	8.0	10	5.0	5.0	12
*Triclosan*	10	14	8.0	7.0	8.0	8.0	13	18	9.0	17	8.0	9.0	8.0	8.0	8.0	8.0	9.0	8.0	8.0
*Warfarin*	8.0	9.0	9.0	9.0	9.0	9.0	9.0	9.0	9.0	9.0	9.0	9.0	8.0	8.0	8.0	8.0	9.0	8.0	8.0

* n.d. = not detected

**Table 6:** Concentration of PPCPs (ng g^−1^) in soil samples (So.n°) of the Albufera Natural Park, Valencia, Spain.

Compound	So.1	So.2	So.3	So.4	So.5	So.6	So.7	So.8	So.9	So.10	So.11	So.12	So.13	So.14	So.15	So.16	So.17	So.20	So.23	So.24	So.25	So.26	So.29	So.30	So.31	So.32	So.33	So.34	So.35	So.41	So.42	So.43	So.44
*Alprazolam*	n.d.*	67	n.d.	67	n.d.	n.d.	n.d.	n.d.	n.d.	n.d.	n.d.	n.d.	n.d.	n.d.	n.d.	n.d.	n.d.	n.d.	n.d.	n.d.	n.d.	n.d.	n.d.	n.d.	n.d.	n.d.	n.d.	n.d.	n.d.	n.d.	n.d.	n.d.	n.d.
*Atenolol*	n.d.	n.d.	n.d.	n.d.	n.d.	n.d.	n.d.	2.0	1.0	n.d.	21	1.0	21	n.d.	1.0	21	n.d.	1.0	21	1.0	n.d.	n.d.	1.0	n.d.	n.d.	n.d.	n.d.	21	n.d.	n.d.	n.d.	n.d.	1.0
*Atorvastatin*	n.d.	n.d.	n.d.	n.d.	n.d.	n.d.	n.d.	n.d.	n.d.	n.d.	n.d.	n.d.	n.d.	n.d.	n.d.	n.d.	n.d.	n.d.	n.d.	n.d.	n.d.	n.d.	n.d.	n.d.	n.d.	n.d.	n.d.	n.d.	n.d.	n.d.	n.d.	n.d.	n.d.
*Bezafibrate*	n.d.	n.d.	n.d.	n.d.	n.d.	n.d.	n.d.	n.d.	n.d.	n.d.	n.d.	n.d.	n.d.	n.d.	n.d.	n.d.	n.d.	n.d.	n.d.	n.d.	n.d.	n.d.	n.d.	n.d.	n.d.	n.d.	n.d.	n.d.	n.d.	n.d.	n.d.	n.d.	n.d.
*Bisphenol A*	9.0	6.0	3.0	9.0	10	4.0	8.0	4.0	7.0	6.0	10	4.0	3.0	4.0	2.0	3.0	2.0	3.0	1.0	2.0	2.0	7.0	2.0	9.0	4.0	n.d.	3.0	15	8.0	9.0	11	7.0	9.0
*Butylparaben*	n.d.	n.d.	n.d.	n.d.	n.d.	n.d.	n.d.	n.d.	n.d.	n.d.	n.d.	n.d.	n.d.	n.d.	n.d.	n.d.	n.d.	n.d.	n.d.	n.d.	n.d.	n.d.	n.d.	n.d.	n.d.	n.d.	n.d.	n.d.	n.d.	n.d.	n.d.	n.d.	n.d.
*Caffeine*	n.d.	22	24	4.0	1.0	24	n.d.	3.0	24	n.d.	22	3.0	2.0	23	1.0	22	1.0	22	3.0	1.0	26	23	23	n.d.	23	1.0	23	3.0	22	16	13	n.d.	2.0
*Chloramphenicol*	1.0	n.d.	n.d.	n.d.	n.d.	n.d.	n.d.	n.d.	n.d.	n.d.	n.d.	n.d.	n.d.	n.d.	n.d.	n.d.	n.d.	n.d.	n.d.	n.d.	n.d.	n.d.	n.d.	n.d.	n.d.	n.d.	n.d.	n.d.	n.d.	n.d.	n.d.	n.d.	n.d.
*Clofibric Acid*	n.d.	n.d.	n.d.	n.d.	n.d.	n.d.	n.d.	n.d.	n.d.	n.d.	n.d.	n.d.	n.d.	n.d.	n.d.	n.d.	n.d.	n.d.	n.d.	n.d.	n.d.	n.d.	n.d.	n.d.	n.d.	n.d.	n.d.	n.d.	n.d.	n.d.	n.d.	n.d.	n.d.
*Codeine*	n.d.	n.d.	n.d.	n.d.	n.d.	n.d.	n.d.	n.d.	n.d.	n.d.	n.d.	1.0	n.d.	n.d.	n.d.	n.d.	n.d.	n.d.	n.d.	n.d.	n.d.	n.d.	n.d.	n.d.	n.d.	n.d.	7.0	n.d.	n.d.	n.d.	n.d.	n.d.	7.0
*Diclofenac*	2.0	3.0	1.0	1.0	n.d.	1.0	1.0	2.0	1.0	n.d.	n.d.	n.d.	n.d.	n.d.	n.d.	2.0	n.d.	2.0	n.d.	n.d.	n.d.	n.d.	n.d.	n.d.	3.0	n.d.	2.0	2.0	n.d.	n.d.	2.0	1.0	n.d.
*Enalapril*	n.d.	n.d.	n.d.	n.d.	n.d.	n.d.	n.d.	n.d.	n.d.	n.d.	n.d.	n.d.	n.d.	n.d.	n.d.	n.d.	n.d.	n.d.	n.d.	n.d.	n.d.	n.d.	n.d.	n.d.	n.d.	n.d.	n.d.	n.d.	n.d.	n.d.	n.d.	n.d.	n.d.
*Ethylparaben*	3.0	n.d.	n.d.	n.d.	2.0	n.d.	n.d.	3.0	n.d.	n.d.	n.d.	n.d.	n.d.	n.d.	n.d.	n.d.	n.d.	n.d.	n.d.	n.d.	n.d.	n.d.	3.0	n.d.	n.d.	2.0	n.d.	n.d.	n.d.	n.d.	n.d.	3.0	n.d.
*Etoricoxib*	n.d.	n.d.	n.d.	1.0	n.d.	n.d.	51	n.d.	n.d.	n.d.	n.d.	n.d.	n.d.	n.d.	n.d.	n.d.	n.d.	n.d.	n.d.	n.d.	n.d.	n.d.	n.d.	n.d.	n.d.	n.d.	n.d.	n.d.	n.d.	n.d.	n.d.	n.d.	n.d.
*Flufenamic Acid*	1.0	n.d.	n.d.	n.d.	n.d.	n.d.	n.d.	n.d.	n.d.	n.d.	n.d.	n.d.	n.d.	n.d.	n.d.	n.d.	n.d.	n.d.	n.d.	n.d.	n.d.	n.d.	n.d.	n.d.	n.d.	n.d.	n.d.	n.d.	n.d.	n.d.	n.d.	n.d.	n.d.
*Furosemide*	n.d.	n.d.	n.d.	n.d.	n.d.	n.d.	n.d.	n.d.	n.d.	n.d.	n.d.	n.d.	n.d.	n.d.	n.d.	n.d.	n.d.	n.d.	n.d.	n.d.	n.d.	n.d.	n.d.	n.d.	n.d.	n.d.	n.d.	n.d.	n.d.	n.d.	n.d.	n.d.	n.d.
*Ibuprofen*	2.0	4.0	10	n.d.	7.0	4.0	3.0	n.d.	3.0	n.d.	n.d.	n.d.	3.0	n.d.	n.d.	n.d.	n.d.	n.d.	n.d.	n.d.	n.d.	7.0	n.d.	n.d.	n.d.	n.d.	8.0	n.d.	4.0	n.d.	76	3.0	n.d.
*Indomethacin*	n.d.	n.d.	n.d.	n.d.	n.d.	1.0	n.d.	n.d.	n.d.	n.d.	n.d.	n.d.	n.d.	n.d.	n.d.	n.d.	n.d.	n.d.	n.d.	n.d.	n.d.	n.d.	n.d.	n.d.	n.d.	n.d.	n.d.	n.d.	n.d.	n.d.	n.d.	n.d.	n.d.
*Lorazepam*	n.d.	n.d.	62	n.d.	n.d.	n.d.	n.d.	n.d.	n.d.	n.d.	62	n.d.	n.d.	62	n.d.	n.d.	n.d.	n.d.	n.d.	n.d.	n.d.	1.0	n.d.	n.d.	n.d.	n.d.	n.d.	n.d.	n.d.	n.d.	n.d.	n.d.	n.d.
*Metformin*	n.d.	n.d.	n.d.	47	47	n.d.	1.0	n.d.	n.d.	n.d.	n.d.	47	n.d.	n.d.	n.d.	n.d.	n.d.	n.d.	n.d.	n.d.	n.d.	n.d.	n.d.	48	n.d.	n.d.	n.d.	n.d.	n.d.	n.d.	n.d.	47	n.d.
*Methylparaben*	n.d.	3.0	2.0	2.0	2.0	3.0	3.0	2.0	2.0	n.d.	n.d.	n.d.	n.d.	n.d.	n.d.	n.d.	n.d.	n.d.	n.d.	n.d.	n.d.	n.d.	4.0	n.d.	n.d.	2.0	n.d.	n.d.	2.0	2.0	2.0	n.d.	n.d.
*Naproxen*	1.0	n.d.	n.d.	n.d.	n.d.	1.0	n.d.	n.d.	n.d.	n.d.	n.d.	n.d.	n.d.	1.0	n.d.	n.d.	n.d.	1.0	1.0	n.d.	n.d.	n.d.	n.d.	n.d.	n.d.	n.d.	n.d.	n.d.	n.d.	n.d.	1.0	n.d.	n.d.
*Omeprazole*	n.d.	n.d.	n.d.	2.0	n.d.	n.d.	n.d.	n.d.	n.d.	n.d.	n.d.	n.d.	4.0	n.d.	4.0	n.d.	n.d.	n.d.	n.d.	n.d.	n.d.	n.d.	n.d.	n.d.	n.d.	3.0	n.d.	n.d.	n.d.	n.d.	n.d.	n.d.	n.d.
*Paracetamol*	n.d.	n.d.	30	n.d.	n.d.	1.0	n.d.	n.d.	30	n.d.	n.d.	n.d.	n.d.	n.d.	n.d.	30	30	31	n.d.	n.d.	30	n.d.	n.d.	n.d.	n.d.	30	n.d.	n.d.	31	n.d.	n.d.	n.d.	n.d.
*Propylparaben*	n.d.	n.d.	n.d.	n.d.	n.d.	n.d.	2.0	n.d.	2.0	n.d.	n.d.	1.0	n.d.	n.d.	1.0	1.0	n.d.	n.d.	n.d.	3.0	n.d.	21	22	n.d.	3.0	n.d.	n.d.	3.0	2.0	2.0	11	4.0	n.d.
*Salicylic Acid*	9.0	n.d.	n.d.	n.d.	2.0	n.d.	n.d.	n.d.	3.0	n.d.	2.0	2.0	20	2.0	6.0	n.d.	5.0	n.d.	2.0	1.0	n.d.	n.d.	n.d.	3.0	n.d.	n.d.	n.d.	6.0	18	n.d.	n.d.	n.d.	5.0
*Simvastatin*	n.d.	n.d.	n.d.	n.d.	n.d.	n.d.	n.d.	n.d.	n.d.	n.d.	n.d.	n.d.	n.d.	n.d.	n.d.	n.d.	n.d.	n.d.	n.d.	n.d.	n.d.	n.d.	n.d.	n.d.	n.d.	n.d.	n.d.	n.d.	1.0	n.d.	n.d.	n.d.	n.d.
*Thiamphenicol*	n.d.	n.d.	n.d.	n.d.	n.d.	n.d.	n.d.	n.d.	n.d.	n.d.	n.d.	n.d.	n.d.	n.d.	n.d.	n.d.	n.d.	n.d.	n.d.	n.d.	n.d.	n.d.	n.d.	n.d.	n.d.	n.d.	n.d.	n.d.	n.d.	n.d.	n.d.	n.d.	3.0
*Tramadol*	n.d.	n.d.	n.d.	2.0	n.d.	1.0	60	n.d.	60	n.d.	n.d.	n.d.	n.d.	n.d.	60	n.d.	60	n.d.	n.d.	n.d.	n.d.	n.d.	n.d.	60	n.d.	1.0	n.d.	n.d.	n.d.	n.d.	60	n.d.	60
*Triclocarban*	1.0	n.d.	n.d.	n.d.	n.d.	n.d.	n.d.	n.d.	n.d.	n.d.	n.d.	n.d.	n.d.	n.d.	n.d.	n.d.	n.d.	n.d.	n.d.	n.d.	n.d.	n.d.	n.d.	n.d.	2.0	n.d.	n.d.	n.d.	n.d.	n.d.	n.d.	n.d.	n.d.
*Triclosan*	4.0	3.0	n.d.	n.d.	n.d.	1.0	n.d.	n.d.	1.0	n.d.	n.d.	n.d.	n.d.	n.d.	n.d.	n.d.	n.d.	n.d.	4.0	n.d.	n.d.	1.0	n.d.	1.0	n.d.	n.d.	n.d.	n.d.	n.d.	5.0	n.d.	n.d.	n.d.
*Warfarin*	1.0	n.d.	n.d.	n.d.	n.d.	n.d.	n.d.	n.d.	n.d.	n.d.	n.d.	n.d.	n.d.	n.d.	n.d.	n.d.	n.d.	n.d.	n.d.	n.d.	n.d.	n.d.	n.d.	n.d.	n.d.	n.d.	n.d.	n.d.	n.d.	n.d.	n.d.	n.d.	n.d.

* n.d. = not detected

**Table 7:** Statistical correlations between the studied pharmaceuticals in **soils** (** Significant correlation at level of P= 0.01. * Significant correlation at level of P= 0.05).

	Aten	Atorv	BZF	BisA	BPN	Caf	CPL	Cod	DFC	Etor	EPB	FlufA	Ibup	IMTN	LZM	Met	MPN	Nap	OPZ	Pmol	PPN	SalA	SVTN	TPL	Tram	TCBN	TCSN
Aten																											
Atorv	.592^⁎⁎^																										
BZF																											
BisA																											
BPN	.417*	.448^⁎⁎^																									
Caf																											
CPL			.726^⁎⁎^																								
Cod																											
DFC																											
Etor																											
EPB							.391*																				
FlufA			.641^⁎⁎^				.915^⁎⁎^																				
Ibup																											
IMTN																											
LZM						.352*																					
Met						−.395*																					
MPN																											
Nap					.437*									.506^⁎⁎^													
OPZ																											
Pmol				−.352*		.349*																					
PPN																											
SalA																			.375*								
SVTN																						.382*					
TPL								.697^⁎⁎^																			
Tram																											
TCBN							.382*		.420*																		
TCSN					.419*		.436*					.482^⁎⁎^															
War			.646^⁎⁎^				.940^⁎⁎^				.446^⁎⁎^	.878^⁎⁎^														.429*	.369*

**Aten**: Atenolol. **Atorv**: Atorvastatin. **BZF**: Bezafibrate. **BisA**: Bisphenol A. **BPN**: Butylparaben. **Caf**: Caffeine. **CPL**: Chloramphenicol. **ClorA**: Clofibric Acid. **Cod**: Codeine. **DFC**: Diclofenac. **Eto**r: Etoricoxib. **EPB**: Ethylparaben. **FlufA**: Flufenamic Acid. **Ibup**: Ibuprofen. **IMTN**: Indomethacin. **LZM**: Lorazepam. **Met**: Metformin. **MPN**: Methylparaben. **Nap**: Naproxen. **OPZ**: Omeprazole. **Pmol**: Paracetamol. **PPN**: Propylparaben. **SalA**: Salicylic acid. **SVTN**: Simvastatin. **TPL**: Thiamphenicol**. Tram**: Tramadol. **TCB**N: Triclocarban. **TCSN**: Triclosan. **War**: Warfarin.

**Table 8:** Statistical correlations between pharmaceuticals in **sediments** (** Significant correlation at level of P= 0.01, * Significant correlation at level of P= 0.05).

	Alpraz	Aten	Atorv	BZF	BisA	BPN	Caf	CPL	ClorA	Cod	DFC	Etor	EPB	FlufA	Furo	Ibup	Met	MPN	OPZ	Pmol	PPN	SalA	SVTN	TPL	Tram	TCBN	TCSN	War
Alpraz																												−.377*
Aten																												
Atorv	.454^⁎⁎^																											
BZF																												
BisA	−.363*	.363*																										
BPN				.371*																								
Caf				.522^⁎⁎^	.375*																							
CPL																												
ClorA																												
Cod							.459^⁎⁎^	.750^⁎⁎^																				
DFC				.365*			.385*		−.337*																			
Etor		.349*				−.516^⁎⁎^	−.366*																					
EPB			.347*		.402*				.329*																			
FlufA		.453^⁎⁎^																										
Furo		.334*			.334*	−.401*				.408*																		
Ibup																												
Met									−.416^⁎⁎^		.363*																	
MPN					.465^⁎⁎^							−.332*	.370*	.330*														
OPZ					.375*											.376*												
Pmol											−.458^⁎⁎^																	
PPN			.469^⁎⁎^						.638^⁎⁎^								−.329*											
SalA	.325*						.572^⁎⁎^		−.561^⁎⁎^		.515^⁎⁎^										−.497^⁎⁎^							
SVTN		.373*						.341*	−.456^⁎⁎^			.393*									−.504^⁎⁎^							
TPL			−.497^⁎⁎^						−.652^⁎⁎^	−.354*			−.330*			.362*	.454^⁎⁎^				−.821^⁎⁎^	.468^⁎⁎^						
Tram		.461^⁎⁎^							−.357*					.830^⁎⁎^										.428^⁎⁎^				
TCSN		.482^⁎⁎^			.374*			.327*						.644^⁎⁎^			.350*							.453^⁎⁎^	.614^⁎⁎^	−.409*		
War	−.377*	.395*	−.425^⁎⁎^		.427^⁎⁎^		.452^⁎⁎^				.339*						.545^⁎⁎^				−.581^⁎⁎^			.492^⁎⁎^		−.581^⁎⁎^	.497^⁎⁎^	

**Alpraz**: Alprazolam. **Aten**: Atenolol. **Atorv**: Atorvastatin. **BZF**: Bezafibrate. **BisA**: Bisphenol A. **BPN**: Butylparaben. **Caf**: Caffeine. **CPL**: Chloramphenicol. **Clor A**: Clofibric Acid. **Cod**: Codeine. **DFC**: Diclofenac. **Etor**: Etoricoxib. **EPB**: Ethylparaben. **Fluf A**: Flufenamic Acid. **Furo**: Furosemide. **Ibup**: Ibuprofen. Met: Metformin. **MPN**: Methylparaben. **OPZ**: Omeprazole. **Pmol**: Paracetamol. **PPN**: Propylparaben. **SalA**: Salicylic acid. **SVTN**: Simvastatin. **TPL**: Thiamphenicol. **Tram:** Tramadol. **TCBN**: Triclocarban. **TCSN**: Triclosan. **War**: Warfarin.

**Table 9:** Statistical correlations between the studied pharmaceuticals in **waters** (** Significant correlation at level of P= 0.01, * Significant correlation at level of P= 0.05).

	Alpraz	Aten	Atorv	BZF	BisA	BPN	Caf	CPL	ClorA	Cod	DFC	Etor	EPB	FlufA	Furo	Ibup	IMTN	LZM	Met	MPN	Nap	Pmol	PPN	TPL	Tram
Aten	.830^⁎⁎^																								
Atorv																									
BZF	.858^⁎⁎^	.899^⁎⁎^																							
BisA	.383*			.374*																					
BPN																									
CPL						.526^⁎⁎^																			
ClorA					.906^⁎⁎^																				
Cod	.871^⁎⁎^	.929^⁎⁎^		.830^⁎⁎^	.423*				.358*																
DFC	.584^⁎⁎^	.598^⁎⁎^		.442^⁎⁎^						.550^⁎⁎^															
Etor	.469^⁎⁎^	.410*		.410*	.532^⁎⁎^				.538^⁎⁎^	.472^⁎⁎^															
FlufA	.908^⁎⁎^	.773^⁎⁎^		.745^⁎⁎^	.454^⁎⁎^				.503^⁎⁎^	.828^⁎⁎^	.530^⁎⁎^	.416*													
Ibup					.422*				.498^⁎⁎^	.372*				.393*	.368*										
IMTN	.356*	.521^⁎⁎^								.589^⁎⁎^	.421*			.450^⁎⁎^		.510^⁎⁎^									
LZM	.870^⁎⁎^	.956^⁎⁎^		.848^⁎⁎^			.380*			.935^⁎⁎^	.624^⁎⁎^	.445^⁎⁎^		.803^⁎⁎^			.456^⁎⁎^								
Met		.477^⁎⁎^								.439*	.476^⁎⁎^							.461^⁎⁎^							
MPN											.376*		.377*												
Nap	.462^⁎⁎^	.609^⁎⁎^		.463^⁎⁎^			.528^⁎⁎^			.593^⁎⁎^	.401*			.550^⁎⁎^	.370*	.395*	.466^⁎⁎^	.586^⁎⁎^	.435*						
Pmol							.874^⁎⁎^														.345*				
PPN					.427*				.554^⁎⁎^		−.418*														
SalA			.354*				.513^⁎⁎^															.572^⁎⁎^			
TPL	.406*	.475^⁎⁎^		.372*						.510^⁎⁎^							.377*	.475^⁎⁎^							
Tram	.951^⁎⁎^	.890^⁎⁎^		.808^⁎⁎^	.401*				.371*	.953^⁎⁎^	.641^⁎⁎^	.514^⁎⁎^		.899^⁎⁎^			.534^⁎⁎^	.924^⁎⁎^	.423*		.546^⁎⁎^			.494^⁎⁎^	
TCBN						.435*			.535^⁎⁎^														.480^⁎⁎^		
TCSN																	.387*								
War	.412*	.386*		.374*	.448^⁎⁎^				.401*	.362*		.473^⁎⁎^		.570^⁎⁎^				.364*							.419*

**Aten**: Atenolol. **Alpraz**: Alprazolam. **Atorv**: Atorvastatin. **BZF**: Bezafibrate. **BisA**: Bisphenol A. **BPN**: Butylparaben. **CPL**: Chloramphenicol. **ClorA**: Clofibric Acid. **Cod**: Codeine. **DFC**: Diclofenac. **Etor**: Etoricoxib. **FlufA**: Flufenamic Acid. **Ibup**: Ibuprofen. **IMTN**: Indomethacin. **LZM**: Lorazepam. **Met**: Metformin. **MPN**: Methylparaben. **Nap**: Naproxen. **Pmol**: Paracetamol. **PPN**: Propylparaben. **SalA**: Salicylic acid. **TPL**: Thiamphenicol. **Tram**: Tramadol. **TCBN**: Triclocarban. **TCSN**: Triclosan. **War**: Warfarin.

**Table 10:** Statistical correlations between pharmaceuticals and intrinsic **soil** characteristics (** Significant correlation at level of P = 0.01. * Significant correlation at level of P = 0.05).

	pH	EC	CO_3_	OM	Na	K	Mg	Ca	CEC
Alpraz	−.380*	.410*			.451^⁎⁎^				
BisA		.358*	.421*	.372*					
Ibup							.353*		
MPN	−.376*			.444^⁎⁎^					
Nap						−.364*			
PPN			−.352*						
TPL		.354*			.446^⁎⁎^				
TCSN									.359*
War		.396*		.414*					

**EC**: Electric conductivity. **CO_3_^=^**: Carbonates. **OM**: Organic Matter. **Na:** Sodium. **K:** Potasium. **Mg:** Magnesium. **Ca:** Calcium. **CEC**: Cation Exchange Capacity.

**Alpraz**: Alprazolam. **BisA**: Bisphenol A. **Ibup**: Ibuprofen. **MPN**: Methylparaben. **Nap**: Naproxen. **PPN**: Propylparaben. **TPL**: Thiamphenicol. **TCSN**: Triclosan. **War**: Warfarin.

**Table 11:** Statistical correlations between pharmaceuticals and intrinsic **sediment** characteristics (** Significant correlation at level of P = 0.01, * Significant correlation at level of P = 0.05).

	OM	CO_3_^=^	Sac	SandT	pH	EC	CEC
Alpraz					−.459^⁎⁎^		
Aten							
Atorv							
BZF							
BisA	.408*						
BPN					.355*		
Caf			−.484^⁎⁎^	.555^⁎⁎^		−.325*	
CPL							
ClorA	.348*	.379*					
Cod							
DFC							
Etor						.384*	
EPB							
FlufA					.378*	−.398*	
Furo		−.382*					
Ibup	−.334*		−.366*	.345*			−.335*
Met							
MPN					.426^⁎⁎^	−.579^⁎⁎^	−.399*
OPZ						−.344*	
Pmol					.348*		
PPN	.512^⁎⁎^	.477^⁎⁎^	.565^⁎⁎^	−.490^⁎⁎^		.510^⁎⁎^	.368*
SalA	−.501^⁎⁎^	−.614^⁎⁎^	−.612^⁎⁎^	.582^⁎⁎^			
SVTN					−.345*		−.328*
TPL		−.586^⁎⁎^	−.470^⁎⁎^	.406*		−.401*	
Tram							
TCSN							
War		−.402*				−.407*	

**OM**: Organic Matter. **CO_3_^=^**: Carbonates. **Sac**: Lime + clay fractions. **SandT**: Total sand fraction. **EC**: Electric conductivity. **CEC**: Cation Exchange Capacity.

**Alpraz**: Alprazolam. **Aten**: Atenolol. **Atorv**: Atorvastatin. **BZF**: Bezafibrate. **BisA**: Bisphenol A. **BPN**: Butylparaben. **Caf**: Caffeine. **CPL**: Chloramphenicol. **Clor A**: Clofibric Acid. **Cod**: Codeine. **DFC**: Diclofenac. **Etor**: Etoricoxib. **EPB**: Ethylparaben. **Fluf A**: Flufenamic Acid. **Furo**: Furosemide. **Ibup**: Ibuprofen. **Met**: Metformin. **MPN**: Methylparaben. **OPZ**: Omeprazole. **Pmol**: Paracetamol. **PPN**: Propylparaben. **SalA**: Salicylic acid. **SVTN**: Simvastatin. **TPL**: Thiamphenicol. **Tram**: Tramadol. **TCSN**: Triclosan. **War**: Warfarin.

**Table 12:** Statistical correlations between the studied pharmaceuticals and intrinsic characteristics of **waters** (** Significant correlation at level of P = 0.01, * Significant correlation at level of P = 0.05).

	pH	T	EC	TDS	Rest	NaCl	DO%	Cl^−^	NO_2_^−^	NO3^=^	SO4^=^	Na	K	Mg	Ca
Alpraz	−.446^⁎⁎^														
Aten	−.414*														
Atorv							.354*								
BisA															
Caf			−.410*			−.365*						−.388*		−.385*	
ClorA				−.355*		−.345*					−.393*				−.361*
Cod	−.458^⁎⁎^														
Enal									.721^⁎⁎^						
EPB							.371*								
FlufA	−.398*														
Furo			−.361*		.381*							−.377*			
IMTN									.473^⁎⁎^						
LZM	−.477^⁎⁎^														
Met										.694^⁎⁎^					
Nap							.359*		.387*					−.480^⁎⁎^	
Pmol		−.354*	−.356*												
SalA		−.402*	−.387*									−.373*			
TPL	−.420*											.364*	.736^⁎⁎^		
Tram	−.491^⁎⁎^														
TCBN					.440*										−.355*

**T**: temperature (°C). **EC**: Electric Conductivity (dS/m). **TDS**: Total Dissolved Solids (mg/L). **Rest**: Resistivity (Ώ). **DO%**: Dissolved Oxygen (%). **Cl^−^**: Chlorides (mg/L). **NO_2_^−^**: Nitrites. **NO_3_^=^**: Nitrates (mg/L). **SO_4_^=^**: Sulfates (mg/L). **Alpraz**: Alprazolam. **Aten**: Atenolol. **Atorv**: Atorvastatin. **Caf**: Caffeine. **ClorA**: Clofibric Acid. **Cod**: Codeine. **EPB**: Ethylparaben. **FlufA**: Flufenamic Acid. **Furo**: Furosemide. **IMTN**: Indomethacin. **LZM**: Lorazepam. **Met**: Metformin. **Nap**: Naproxen. **Pmol**: Paracetamol. **SalA**: Salicylic Acid. **TPL**: Thiamphenicol. **Tram**: Tramadol. **TCBN**: Triclocarban.

## 2 Experimental Design, Materials and Methods

The water characteristics were in situ measured using a portable Multiparameter Eutech Instrument CyberScan PCD 650 (Thermo Fisher Scientific, Basel, Switzerland). The soil and sediment characteristics were established in the laboratory using standard procedures. Organic matter was determined by oxidation with dichromate [5]. Carbonate was determined using the Bernard calcimeter [6] method and cationic exchanger capacity was calculated measuring sodium, potassium, magnesium and calcium by extraction with 1 M ammonium acetate solution and inductively coupled plasma optical emission spectroscopy) (ICP-OES) following the method of Rhoades [7]. Electric conductivity and pH were determined in the soil saturation extract with a pH-meter according to Richards [8] and finally, % of lime, clay and sand were established using an hydrometer according to the Bouyoucos [9] method. The waste and surface waters (200 mL) were vacuum filtered by a 0.6-µm glass fiber filter (GA-55, 90 mm - Advantec MFS, Dublin, CA, USA) and stored at -20°C until the analysis. The sediments were lyophilized with a Virtis lyophilizer (SP Scientific, Gardiner, NY, USA) and the soil samples were sieved, and air-dried in the dark at 20°C to reduce the moisture content. The samples of water, soil and sediment were spiked with labeled internal standards to quantify the PPCPs present in the all environmental compartments. A previous solid-liquid extraction was performed for solid samples (1g), with the use of 15 mL of a mix containing Milli-Q water, McIlvaine–EDTA buffer and methanol (MeOH) in equal parts. The mixture was homogenized for 5 min by vortex agitation, sonicated for 10 min, and centrifuged for 6 min at 3000 rpm and 10°C. The supernatant was separated and diluted with Milli-Q water to 200 mL. Then, the dilution was treated such as water extraction procedure.

The clean-up that involves PPCPs isolation and concentration were performed by Solid Phase Extraction (SPE). Two methods employing different cartridges Strata-X and Strata-X-CW (Phenomenex, 33 µm, 200 mg/6 mL) characterized by a *polymeric reversed* and *polymeric weak cation-exchange* stationary phase, respectively, were used. Strata-X was activated with 6 mL MeOH, 6 mL Milli-Q water and with 6 mL 2mM *Sodium Dodecyl Sulphate* (SDS) *solution* (**SDS method**). While, Strata-X-CW was activated without SDS solution (**WC method**). The analytes were eluted with 6mL of MeOH and 3 mL of MeOH–DCM (50:50 v/v) for reversed phase and with 6 mL of MeOH–NH_4_OH (95:5 v/v) for weak cation-exchange phase by gravity. The eluates were evaporated at 40°C and redissolved to 1 mL with mobile phase before injection.

Alprazolam, atorvastatin, caffeine, chloramphenicol, diclofenac, flufenamic acid, furosemide, ibuprofen, omeprazole, paracetamol and thiamphenicol were detected by *WC method* in water, sediment and soil matrix. While, atenolol, bezafibrate, butylparaben, clorfibric acid, enalapril, ethylparaben, etoricoxib, indomethacin, lorazepam, metformin, methylparaben, naproxen, propylparaben, salicylic acid, simvastatin tramadol, triclocarban, triclosan, warfarin by *SDS method*. Only bisphenol A and codeine were determined by both methods, *SDS* in water matrix and *WC* in sediment and soil.

Instrumental analysis was performed by 1260 Infinity UHPLC (ultra-high-performance liquid chromatography) system coupled to mass spectrometry with a triple quadrupole mass detector (6410 QqQ-MS) from Agilent Technologies (Santa Clara, CA, USA). The electrospray ionization (ESI) was applied in negative and positive mode. The mobile phase consisted of MeOH (solvent A) and water (solvent B) both with NH_4_F at 2.5 mmol L^−1^ for negative mode and MeOH (solvent A) and water (solvent B) with 0.1% formic acid in both solutions for positive mode. The calibration curves used to quantify the environmental contaminants were prepared in H_2_O-MeOH (70-30) and in solvent with SDS to obtain correct quantification of those compounds.

The qualitative and quantitative analysis of each chromatogram were performed by MassHunter Workstation (version 10.0 Software) supplied by Agilent Technologies. The statistical relationship of the different contaminants between them and with the environmental parameters in the three matrices selected (water, soil and sediment) was carried out by Statistical package IBM SPSS (version 26.0).

The limits of detection (LODs) and limits of quantification (LOQs) were estimated experimentally, spiking blank samples, at the lowest concentration (10 ng g^−1^ and 50 µgL^−1^), with the PPCPs pre-extraction and estimating the analyte concentration able to provide a signal-to-noise ratio of 3 and 10 respectively. The LODs ranged from 1.65 and 16.65 ngL^−1^ in waste and surface water, and from 0.33 and 6.67 ng g^−1^ dry weight (d.w.) in sediment and soil. Particularly, to established the matrix effects (ME) two eight point calibration curves (10, 25, 50, 75, 100, 250, 500, 1000 ng/mL) were compared: (i) one prepared in solvent (that is H_2_O-MeOH (70-30) or this mixture with SDS depending on the method) and (ii) the other prepared in blank matrix extract, also redissolving the extract in H_2_O-MeOH (70-30) or this mixture with SDS. Then, the ME was calculated according to the formula:

(1) $$ME\;\left(\%\right)=\left(\frac{Slope\;of\;calibration\;curve\;in\;matrix}{Slope\;of\;calibration\;curve\;in\;solution}-1\right)\times 100$$

This information together with other experimental information were described in the Sadutto [2] work.

## Declaration of Competing Interest

The authors declare that they have no known competing financial interests or personal relationships, which have, or could be perceived to have, influenced the work reported in this article.
